# Digital Technologies That Support Meaningful Connections in Care Homes: Scoping Review

**DOI:** 10.2196/88181

**Published:** 2026-03-03

**Authors:** Deborah Muldrew, Rosemary Bradley, Kelly Conway

**Affiliations:** 1 Institute of Nursing and Health Research University of Ulster Belfast United Kingdom; 2 Institute of Nursing and Health Research University of Ulster Londonderry United Kingdom

**Keywords:** scoping review, digital technology, meaningful connection, social connection, care homes, nursing homes, older people, aged

## Abstract

**Background:**

Meaningful connections in which people feel valued, seen, and heard are essential for social health and well-being. However, individual, systemic, and structural barriers exist within care homes that exacerbate risks for social isolation in this population. Leveraging digital technology to promote meaningful connections has the potential to affect positive health outcomes; however, the evidence base within the care home environment is not clear.

**Objective:**

This scoping review aimed to identify how digital technologies are used in long-term care settings to facilitate meaningful connections.

**Methods:**

Following Joanna Briggs Institute and PRISMA-ScR (Preferred Reporting Items for Systematic Reviews and Meta-Analyses extension for Scoping Reviews) reporting guidelines, 6 online databases (CINAHL, MEDLINE, PsycINFO, Scopus, IEEE Explore, and ACM) were searched on December 12, 2025. Studies were included if they were published in English; included care home residents, relatives, or staff; and directly discussed the application of a digital technology that focused on building meaningful connections. No date limiters were applied. Gray literature was searched through Google Scholar and conference proceedings, and forward and backward citation searching was also undertaken. Papers were managed in Covidence and reviewed against inclusion criteria by 3 reviewers (DM, RB, and KC). Results were presented using tabular, graphical, and narrative summaries to describe how the findings relate to the review objectives.

**Results:**

In total, 72 studies were included. More than half of the studies were published since 2022 (38/72, 53%). Australia (15/72, 21%) and the Netherlands (12/72, 17%) had the highest frequency of publications. Technologies including robotics, virtual reality, mobile or tablet apps, digital devices, and online programs have been used in care homes. Factors underpinning the development of meaningful connections include getting to know the person, increases autonomy and choice, source of enjoyment and fun, facilitates communication, and builds community. Engagement, well-being or satisfaction, emotional response, quality of life, purpose and meaning, social closeness, loneliness, depression and anxiety, and psychosocial capacity were the main indicators of meaningful connections. Limitations include English-language restriction, potential missed studies on social connection, exclusion of passive technologies, and heterogeneous outcome measures.

**Conclusions:**

This review is the first to explore how digital technologies are applied in care homes to facilitate meaningful connections and identify how digital technology is a catalyst for meaningful human connection rather than a replacement. The application of generative artificial intelligence technology is currently a gap in the evidence base and needs to be considered in conjunction with key stakeholders to ensure that future developments meet the social needs of the entire community. Future research needs to consider how new evaluation metrics can be developed that combine digital engagement data with validated measures of meaningful connection to assess the person-centered impact of generative artificial intelligence technologies in care homes.

## Introduction

Social connection describes how individuals relate to each other, encompassing both observable aspects such as engagement with social networks and the experiences of loneliness or social connectedness [1]. People who are socially connected feel an overall sense of belonging to a group or community. The World Health Organization (WHO) recognizes social connection as a global public health priority. Unfortunately, societal trends over a number of decades indicate that, as a population, we have become less socially connected, and a high percentage of the population is lonely [2]. Current global estimates suggest that 1 in 4 older adults experience social isolation [3]. This concern is likely to become more prevalent, given the speed of population aging, which is significantly faster than in previous decades. Current population predictions by the WHO estimate that by 2050, the number of individuals aged 60 years and older will double to 2.1 billion globally, and the number of individuals aged 80 years and older will triple to 426 million [4]. An aging population, coupled with societal shifts in how care is delivered, is predicted to place a substantial rise in the demand on long-term care facilities [5]. Long-term care consists of a range of medical or nursing care, personal care (help with activities of daily living), assistance services (help with instrumental activities of daily living), and support to maintain social relationships and activities [6]. The term “care homes” encompasses 2 types of long-term care settings: residential care homes (for people requiring personal care) and nursing homes (for people requiring personal and nursing care) [7]. Individual, systemic, and structural barriers exist within care homes that exacerbate the risks for social isolation in this population [8].

While the goal toward social connection is well documented, meaningful connections are the first step toward creating an enriching life and can have a significant impact on health and well-being [9]. Meaningful connections are characterized by deeper, more intimate relationships that make the person feel seen, heard, and valued. For the purpose of this research, we have defined meaningful connections as “interpersonal interactions, whether one-off or repeated, that foster feelings of being seen or heard, empathy, or enhanced social belonging; and/or lead to indicators such as reduced loneliness, increased engagement, or improvements in psychosocial outcomes.” An avenue to such meaningful connection is meaningful activity. National Institute for Health and Care Excellence guidelines recommend that people should be offered opportunities to participate in meaningful activity that promotes health and mental well-being [10]. Meaningful activities are defined as “all activities or occupations that are significant or meaningful for the person and reflect someone’s current and past interests, routines, habits, and roles and are adjusted to someone’s abilities” [11]. Engagement in personally meaningful activities leads to improved psychological well-being [12], and research into people with dementia living in care homes reported improvements in behavioral and psychological symptoms through the provision of meaningful activities [13]. However, when the focus becomes the activity itself and not the meaningful connection formed between the people involved, this approach could lead to task-oriented approaches to care, currently being challenged within health care [14]. Furthermore, activities viewed as something separate from everyday routine, and require additional staffing to coordinate in a busy environment where resources and staffing are already overstretched, are less likely to be meaningful or person-centered [15]. Instead, considerations should be given to the meaningful connections that can form between people at any time, not just during planned or purposeful meaningful activity.

According to Kemp et al [16], meaningful connections are essential for well-being and may serve as effective nonpharmacological approaches to mitigate adverse behavioral and psychological manifestations such as anxiety, depression, apathy, agitation, and aggression. Gonnord et al [17] also demonstrated that engagement could positively affect a number of aspects of well-being, such as social connection, personal autonomy, physical health, and mental stimulation. Social connectedness and participation in meaningful activities are important sources of joy, which have been shown in the literature as important to the health and social care of older people [18]. In the nursing home context, meaningful social relationships with peers and families foster a sense of belonging, purpose, achievement, continuity, and significance, contributing to their psychosocial well-being [19]. Within nursing homes, social connectedness may be an important modifiable risk factor for adverse health outcomes [20]. However, barriers to quality social participation exist at an individual level, for example, personal motivations and preexisting social networks, and at a community level, including accessibility and neighborhood cohesion [21]. Although these findings come from research into older adults more widely, their relevance to older adults living in care homes is clear. As highlighted by Nygaard et al [22], there are additional obstacles and challenges to achieving person-centered meaningful connections in care homes including a regimented structure to care home life and a lack of time for staff to spend quality time with individual residents, as well as a lack of connectedness within meaningful social relationships.

Leveraging digital technology to promote social connectedness has the potential to affect positive health outcomes [23]. The introduction of digital technologies was accelerated into care homes as a result of global restrictions during the COVID-19 pandemic, despite reported systemic and structural barriers [24]. Technology-supported interventions can improve old-age social well-being among older adults living in social isolation [25]; however, the evidence base is typically condition-specific (eg, dementia) or focused on treating a clinical outcome (eg, depression), rather than exploring the role of technology to facilitate meaningful connections among residents, relatives, and staff. Furthermore, much of the evidence based around digital technology considers technology solely for passive monitoring purposes such as digital movement sensors or blood pressure monitors. Therefore, a review of the evidence is required to fill the current evidence gap on how digital technologies can be used to form meaningful connections in care homes. Given the overall limited focus within the evidence base on application of digital technology to support meaningful connections among the wider care home population, formulating a precise question for a systematic review was not possible. Instead, a scoping review supported the mapping of all digital technologies applied to the care home setting with the aim of supporting meaningful connections [26]. A preliminary search of CINAHL, MEDLINE, and *JBI Evidence Synthesis* was conducted, and no current or underway systematic reviews or scoping reviews on the topic were identified.

The aim of this scoping review is to identify how digital technologies are used in long-term care settings to facilitate meaningful connections. The objectives related to this aim are (1) to identify the scope of digital technologies being applied within the care home setting to build meaningful connections, (2) to understand the factors through which digital technologies are able to support meaningful connections, (3) to identify which outcomes are measured to evidence a change in meaningful connections, and (4) to identify gaps in the evidence base to inform future research, policy, and practice.

## Methods

### Design

A scoping review methodology following guidance from the Joanna Briggs Institute (JBI) and PRISMA-ScR (Preferred Reporting Items for Systematic Reviews and Meta-Analyses extension for Scoping Reviews; Multimedia Appendix 1) [27] reporting guidelines was selected as the most appropriate evidence synthesis approach, based on the guidance from Munn et al [26]. The aim was to scope and map the existing literature on digital technologies used in long-term care homes to support meaningful connections, including technologies used, factors showing how the digital technologies promote meaningful connections, and outcome measures of such connections. The systematic approach outlined by the JBI [28] was chosen for this review to provide a high degree of rigor and transparency, to ensure that the review can be replicated easily, and to increase its value to health care decision-makers. Adherence to the items reported in the PRISMA-S (Preferred Reporting Items for Systematic Reviews and Meta-Analyses Literature Search Extension) checklist was used to strengthen the transparency of reporting the search [29]. Ethics approval was not required because scoping reviews synthesize existing publicly available literature without involving human participants or identifiable data.

### Protocol

A protocol for the scoping review was developed and subject to peer review within the first author’s (DM) higher education institution. However, this protocol was not registered externally. Following the review of the protocol, changes to the final review included the addition of “aged care” as a concept, the addition of 2 databases (ACM Digital Library and IEEE Xplore), and the expansion of the gray literature searches to include a range of relevant conference proceedings from 2025.

### Eligibility Criteria

As the scoping review question guides and directs the development of the inclusion criteria, the JBI recommends the use of the population, concept, and context mnemonic in ensuring the eligibility of the primary research question [28]. The research questions were developed through consultation with the research team and key stakeholders focusing on the overall aim of the scoping review. Textbox 1 presents the identified population, concept, and context and the primary research question.

**Population, concept, and context framework for defining the inclusion criteria of studies.**

**Population**

Participants include care home residents, relatives, and staff. Care homes include residential and nursing homes. Studies were excluded if they were undertaken in nonresidential (day care) services, supported housing, facilities for people with learning disabilities or enduring mental health conditions, or other community care settings such as in the person’s own home.

**Concept**

Meaningful connections, activities, or engagement as a goal of the digital technology. Studies were excluded if they focused solely on clinical health or other outcomes without data related specifically to meaningful connections.

**Context**

Digital technologies used actively with or by people in care homes. Studies were excluded if the technology was solely for passive monitoring purposes, such as digital movement sensors or blood pressure monitors, or did not prioritize the creation of meaningful connections through digital means.

**Research question**

How do digital technologies facilitate meaningful connections in care homes?

Studies published in English were included due to practical limitations (translator not available to the research team). No date limitations were applied to the searches to allow for an exploration of how digital technologies have changed over time in type and frequency of application in care homes. No published search filters were used. This scoping review included all primary research, including qualitative, quantitative, and mixed methods approaches. Opinion papers and editorials were also considered for inclusion in this scoping review; however, they were required to report some primary data.

### Information Sources

CINAHL Ultimate (EBSCOhost), MEDLINE (Ovid), Scopus, ACM Digital Library, IEEE XPlore, and PsycINFO (Ovid) were independently searched on December 12, 2025. Gray literature was searched using Google Scholar (first 100 hits) and websites including the WHO and National Institute for Health and Care Excellence. Published conference proceedings from 2025 were hand-searched to identify any new publications that may not be published and accessible through traditional database searches. Conferences included the CHI Conference on Human Factors in Computing Systems, Good IT Proceedings of the 2025 International Conference on Information Technology for Social Good, COMPASS ‘25: Proceedings of the 2025 ACM SIGCAS/SIGCHI Conference on Computing and Sustainable Societies, C&C ‘25: Proceedings of the 2025 Conference on Creativity and Cognition, MuC ‘25: Proceedings of the Mensch und Computer 2025, WWW ‘25: Companion Proceedings of the ACM on Web Conference 2025, and VRST ‘25: Proceedings of the 2025 31st ACM Symposium on Virtual Reality Software and Technology.

### Search Strategy

The search strategy aimed to locate both published and unpublished studies. A 3-step search strategy was used in this review. First, an initial limited search of MEDLINE Ovid and CINAHL Ultimate was undertaken to identify papers on the topic in conjunction with a university librarian and subject specialist. This search was based on the subject expertise of the team and the database searching skills of the librarian. The text words contained in the titles and abstracts of relevant papers and the index terms used to describe the papers were used to develop a full search strategy (Multimedia Appendix 2).

The search strategy, including all identified keywords and index terms, was adapted for each included database and information source. A broad range of keywords and MeSH (Medical Subject Headings) headings were used to reduce the likelihood of missing relevant literature, and then, the inclusion and exclusion criteria were applied strictly to ensure that the papers included were relevant to the objectives. Forward and backward citation searching on the included sources of evidence was undertaken to identify additional studies. No authors were contacted, as all papers identified through the search strategy were available. Email alerts were set up for each database to provide weekly updates of new literature.

### Study Selection

Following the search, all identified citations were collated and uploaded into Covidence, and duplicates were removed. Duplication was managed within Covidence by the in-built duplication feature and thorough review from the authors. Studies were sorted alphabetically by author to allow for identification of additional duplicates missed by Covidence. Following a pilot test, titles and abstracts were screened by multiple independent reviewers (DM, KC, and RB) for assessment against the inclusion criteria for the review. Potentially relevant sources were retrieved in full and stored in Covidence. The full text of selected citations was assessed in detail against the inclusion criteria by multiple independent reviewers (DM, KC, and RB). Reasons for exclusion of sources of evidence at full text that did not meet the inclusion criteria were recorded and reported in the scoping review. Any disagreements that arose between the reviewers at each stage of the selection process were resolved through discussion.

### Data Charting

Data extraction was undertaken to develop a logical and descriptive summary of the results, aligned to the objectives of the review. Data were extracted from papers included in the scoping review by multiple independent reviewers (DM, KC, and RB) using a data extraction tool developed by the research team. It was based on the JBI data extraction template to meet the objectives of the review. Each researcher (DM, KC, and RB) piloted the data extraction tool on 5 papers before meeting to discuss the utility of the tool. Originally, all findings were collected in one column; however, this was leading to confusion, and key data being overlooked. The data extraction tool was updated to have 3 separate results columns: one for the type of digital technology discussed, one for the factors that create meaningful connections, and one for the outcome measures identified. Any disagreements that arose between the reviewers were resolved through discussion. Once data extraction was completed for the included studies, a second reviewer (RB) repeated the data extraction process for 10% (7/72) of the included studies to ensure that all relevant information was extracted accurately. See Multimedia Appendix 3 for the completed data extraction table.

### Data Items

The data extracted included specific details about the author, year of publication, country of origin, population and sample size, methods, type of technology used, factors supporting meaningful connection, and outcome measures.

### Quality Appraisal

Quality appraisal was not undertaken, aligned to methodological norms for scoping reviews. However, a descriptive assessment of each study design, based on the levels of evidence identified by Melnyk and Fineout-Overholt [30], was included to support the development of more robust policy and practice recommendations. The rating scale ranged from 1=strong (systematic review or meta-analysis of randomized controlled trials) to 7=weak (opinion pieces and narrative literature review). In total, 65 studies were categorized as level 6, representing evidence from a single qualitative or descriptive study.

### Synthesis of Results

An overview of included studies was used to summarize bibliographic information. A narrative summary was developed to describe how the findings relate to the core objectives of the review: the digital technologies used, the factors through which meaningful connections are established, and the outcome measures reported. Three independent researchers (DM, KC, and RB) read and reread the included studies and extracted data relevant to the core objectives. The extracted data were then synthesized to ensure that a comprehensive account of relevant information was presented in the final data extraction table. A narrative summary accompanying the data presented was developed by the first author (DM) and reviewed and edited by the second and third authors (RB and KC).

## Results

### Selection of Sources of Evidence

**Figure 1:**
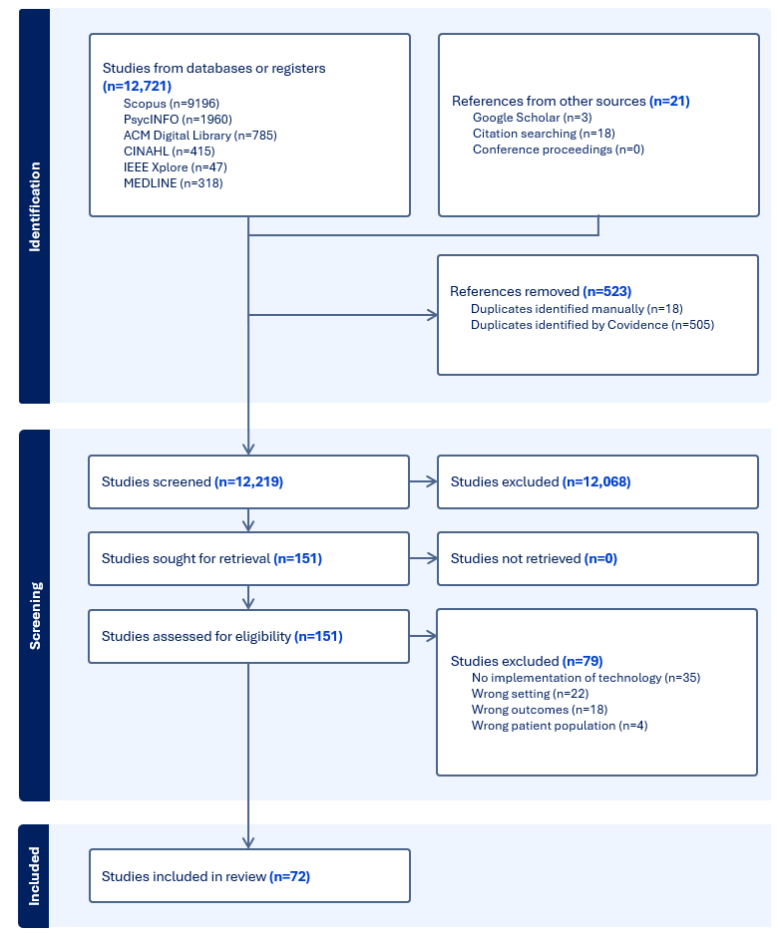
PRISMA (Preferred Reporting Items for Systematic Reviews and Meta-Analyses) flow diagram.

In total, 12,742 papers were identified through the database searches, citation searching, and gray literature. Following the removal of duplicates, 12,219 papers were screened against the inclusion and exclusion criteria. The results of the search are shown in the PRISMA flow diagram (Figure 1).

In total, 151 full-text studies were assessed for eligibility, and 79 were excluded at this point. Reasons for exclusion included no digital technologies being implemented in the paper (35/79, 44%), papers focused on clinical outcomes instead of meaningful connection (18/79, 23%), research was undertaken in the wrong setting (22/79, 28%), or papers included a range of settings or populations and did not have a separate analysis available for the focus of the review (4/79, 5%). In total, 72 studies were identified for inclusion in the final review (Multimedia Appendix 3).

### Characteristics of Included Studies

The first study that reported on digital technologies to support meaningful connections in care homes was published in 2002; however, more than half of the studies were published since 2022 (38/72, 53%; Table 1). While older studies focused more on film screenings and videophones, virtual reality (VR) and digital apps were the most popular digital technologies in 2024-2025, showing a change over time in the types of digital technologies explored.

**Table 1:** Year of publication.

Year	Values, n (%)
2002	1 (1)
2003	1 (1)
2009	1 (1)
2010	1 (1)
2013	1 (1)
2014	1 (1)
2016	2 (3)
2017	1 (1)
2018	3 (4)
2019	4 (6)
2020	9 (13)
2021	10 (14)
2022	11 (15)
2023	7 (10)
2024	14 (19)
2025	5 (7)

In total, 15 studies were undertaken in Australia, 12 studies in the Netherlands, 8 studies in Canada, 5 studies in Norway and the United Kingdom, respectively, 4 studies in the United States, 3 studies across multiple countries, 3 studies in Germany, Italy, and Taiwan, respectively, 2 studies in Denmark, Finland, and Sweden, respectively, and 1 study in Egypt, Ireland, Japan, Jordan, and Switzerland, respectively. Study settings were identified as care homes (17/72, 24%), residential homes (17/72, 24%), nursing homes (13/72, 18%), long-term care settings (16/72, 22%), and other care facilities including “aged care residences” (9/72, 13%). Participants included care home residents (47 studies, 1432 residents), professional caregivers (30 studies, 811 people), and friends and family (18 studies, 407 people) primarily. Some studies included other key stakeholders alongside residents, families, and staff, including high school students, expert older adults, and volunteers. In total, 17 studies specifically included patients with dementia in their sample, including a total of 269 residents with a dementia diagnosis.

### Types of Digital Technologies

Types of digital technologies used in care homes to support meaningful connections were grouped into 5 main categories: mobile and tablet apps, VR and immersive technology, robotics, digital devices and ambient technology, and online or computer-assisted programs (Table 2).

**Table 2:** Summary of intervention types and functions.

Intervention and function			Examples
**Mobile and tablet apps (n=18)**			
	Communication	Ticket To Talk app iPad communication app (unnamed) Video calling (n=4; eg, FaceTime) Teleconferencing (n=2)	
	Reminiscence	MemoryKeeper app StoryTiling app	
	Social activity	Connecting Today app (remote visiting) A Better Visit app (shared activity) Cognitive stimulation therapy app (n=2) Virtual visits to places of personal significance (n=2) iBeacons and audio server with iOS applications (virtual museum visit; n=2)	
**Devices and ambient technology (n=21)**			
	Wearables and sensory stimulation	MEMENTO wearable reminiscence technology Hepatic devices during video calls (warm hand, vibrating music strap, weighted collar, warm hug robot, Hey bracelet) Interactive sound cushion “VITA” (n=3) Alternative controller musical instrument	
	Creative engagement	VENSTER interactive art (n=2) ABBY interactive touchscreen AmbientEcho interactive media system Vodafone smart photo frame	
	Social activity	Computer kiosks Digital television for reminiscence Interactive table Motitech bikes KOMPa “one button computer connecting generations” (n=4) SAMb companions	
	Smart home and voice-controlled assistants	Google Nest Hub Max Google Home Assistant	
**Online and computer-assisted programs (n=8)**			
	Reminiscence	Videotape (Remembering When) Film screenings Computer Interactive Reminiscence and Communication Aid	
	Social activity	SitDance (interactive touchscreen with exercise program) Virtual creative aging program (museum and music) ArtOnTheBrain (museum)	
	Communication	Online social networking Internet-based videoconferencing	
**Virtual reality (VR) and immersive technology (n=10)**			
	Social activity	VR treadmill VR Take A Cycle With Me VR cycling with pedal exercisers and GoPro video footage VR Vacation HomeImmersive 3D VR with hands-on horticultural therapy VR companion Kiera	
	Reminiscence	3D virtual worlds (reminiscence room, virtual tours, gardening) VR reminiscence with VR photo albums or videos SENSE-Garden (n=2)	
**Robotics (n=14)**			
	Social activity	LiveNature (sheep) robotic social sheep (n=3) Robotic pets (cat and dog; n=2) Paro (baby harp seal; n=2) Zora humanoid Adam humanoid Matilda Pepper semihumanoid Rebecca social robot Zoomorphic cleaning robot Robot-facilitated dance therapy	

^a^KOMP: a communication tool developed by Norwegian company No Isolation, designed to bridge the gap between older adults and their digitally experienced families. It consists of a screen with one single button that turns it on and off and an app that is connected to the screen.

^b^SAM: an interactive companion that plays sounds and displays colors in response to touch-based inputs and can mimic or react to the input of a paired device to stimulate social engagement between care home residents.

Mobile apps were discussed in 18 studies [31-48]. The primary aim of these interventions was to increase communication (eg, Ticket to Talk, video calling, and FaceTime), support reminiscence (eg, MemoryKeeper and StoryTiling), increase access to shared activities (eg, A Better Visit, Virtual Museum visiting app paired with iBeacons and audio servers, cognitive stimulation therapy, and virtual visits), and deliver cognitive stimulation therapy. Digital devices and ambient-assisted living technologies were reported in 21 studies [49-69]. New devices included wearables and sensory stimulation such as MEMENTO wearable reminiscence technology and hepatic devices to wear during video calls, creative engagement devices such as VENSTER interactive artwork and AmbientECHO interactive media system, social activities such as Motitech bikes and interactive tables, and smart home and voice-controlled assistants such as Google Nest Hub Max. Online and computer-assisted programs were reported in 8 studies [70-77]. These programs supported reminiscence such as videotapes, film screenings, and computer-interactive reminiscence-specific programs, social activity such as interactive exercise videos (SitDance), creative aging programs (museum visits and music), and communication such as online social networking. VR and immersive experiences were reported in 10 studies [78-87], including social activities such as VR treadmills, VR cycling (Take a Cycle with Me), VR Vacation Home, a VR companion (Kiera), an immersive personalized sensory experience (SENSE-Garden), and reminiscence activities such as 3D virtual words with a reminiscence room and VR reminiscence through virtual photo albums and videos. The application of robotics in care homes was reported in 14 studies [88-101], including robotic animals (sheep, cats, dogs, and seals), humanoids and semihumanoids (Adam, Matilda, Pepper, Rececca, and Zora), a zoomorphic cleaning robot with a social role, and robot-facilitated dance therapy. One study reported on the application of multiple digital technologies including digital music therapy, mobile apps, video games, videoconference, social robots, VR, and other technologies [102].

### Outcomes to Measure Meaningful Connection

In total, 38 studies primarily used qualitative analysis techniques such as thematic analysis to assess outcomes, and another 12 studies combined qualitative analysis techniques with quantitative analysis. Observation was also a commonly used technique to observe changes in meaningful connections such as observing verbal and nonverbal responses, enjoyment, ease of responding to stimuli, and eye contact [35,72,88,99]. Engagement, well-being or satisfaction, emotional response, quality of life, purpose and meaning, social closeness, loneliness, depression and anxiety, and psychosocial capacity were the main themes identified from the scales used to indicate whether meaningful connections were present (Table 3). Studies also considered technology acceptance and clinical outcomes such as cognitive function, activities of daily living, and neuropsychiatric symptoms.

**Table 3:** Summary of outcome measurement instruments.

Instrument		Studies	Focus	Commentary on the tool from the authors
**Engagement**				
	Engagement of a Person With Dementia Scale	[94]	Evaluates user engagement through activity participation (affective, visual, verbal, behavioral, and social).	Multiple raters can be a strength; however, when staff are unfamiliar with using the tool, and different staff complete the tool due to shift changes, this may lead to inconsistencies in the results.
	Observational Measurement of Engagement	[88,94,95]	Direct observation measure of activity engagement for persons with dementia (attention and attitude).	Rated based on direct observations on-site by 2 different raters.
	Music in Dementia Assessment Scale (adapted)	[70]	Trained raters observe and quantify behaviors (interest, response, initiation, involvement, and enjoyment).	No comment.
	Engagement in Meaningful Activities Survey	[70]	Measures the impressions of the meaningfulness of daily activities.	Some of the items focus on domains typically impacted by aging and may influence responses.
	Video Coding Incorporating Observed Emotion	[51]	Overview of emotional responses through 6 dimensions of engagement (emotional, verbal, visual, behavioral, collective engagement, and agitation).	A second coder is required to enhance the reliability and validity of the analysis. Automatic interpretation of affective facial expression and qualitative measures of connectedness and social inclusion should be included.
	Ethographic and Laban-Inspired Coding System of Engagement	[88]	Measures engagement of a person with dementia through observational behaviors (head, torso, and hands or arms).	Instructions are needed to ensure consistency in the measurement procedures (eg, reduce personal conversations irrelevant to the study).
	Visual Focus of Attention	[103]	OpenVINO used to autonomously track 2D positions of users’ eyes, ears, and nose.	No comment.
**Well-being and satisfaction**				
	Warwick-Edinburgh Mental Well-Being Scale	[59]	Unidimensional self-report instrument (14 items) assessing well-being administered either in person or via telephone.	Previous studies demonstrated the feasibility of the tool’s use with vulnerable populations.
	World Health Organization-Five Well-Being Index	[81]	Self-report measure of mental well-being.	Previous studies demonstrated validity in screening for depressive mood.
	Satisfaction With Life Scale	[75]	Self-report 5-item scale assessing global satisfaction with life.	No comment.
	Ryff’s Psychological Well-Being Scale	[85]	Six dimensions—autonomy, environmental mastery, personal development, supportive relationships, purpose in life, and acceptance of oneself.	No comment.
**Emotional response**				
	Observed Emotional Rating Scale	[94]	Measures the extent of emotional expressions (pleasure, anger, anxiety, sadness, and general alertness).	Rated based on direct observations on-site by 2 different raters. Evidence supports interrater reliability for each state and concurrent and discriminant validity for use by trained raters.
	Four items measuring enjoyment from the Game Experience Questionnaire	[40]	Self-report 4 items measuring enjoyment in the experience.	No comment.
	People Environment Apathy Scale—Apathy subscale	[94]	Four-item Likert scale assessing apathy-related behaviors through facial expression, eye contact, physical engagement, purposeful activity, verbal tone, and verbal expression.	Valuable tool to assess the extent of intrinsic motivation to behave despite being positively or negatively engaged.
**Quality of life**				
	EuroQOL^a^ EQ-5D-5L	[80]	A 2-page instrument with a descriptive system and visual analog scale, comprising 5 dimensions (mobility, self-care, usual activities, pain or discomfort, and anxiety or depression).	Valid measure of health-related quality of life in people living in residential care.
	Multicultural Quality of Life Index	[70]	Captures subjective quality of life over 10 dimensions such as physical well-being and spiritual fulfillment.	Two domains did not apply to long-term care residents, so were removed.
	Quality of Life in Late Stage Dementia scale	[70]	Rates observed resident behaviors on 11 items such as crying or appearing uncomfortable over the last week.	No comment.
	DEMQOL^b^ Proxy instrument	[38]	A 31-item scale completed by proxy to measure quality of life—social relationships items selected (getting in touch with people, having enough company, and being able to help other people).	Evidence supports the DEMQOL Proxy’s internal consistency and test-retest reliability when used with people living with mild to moderate and severe dementia, and its content, convergent, and discriminate validity.
	Quality of Life—Alzheimer’s Disease	[67]	A 13-item questionnaire used to provide a self-reported indication of quality of life.	Multiple assessors may have introduced sources of variability.
**Purpose and meaning**				
	Chinese Health Questionnaire	[86]	A 12-item scale measuring both participant health and mattering (the degree of being valued by significant others), meaning of life, loneliness, and depression.	Valid tool for use in older adults.
	Purpose in Life Survey	[86]	Nine items (Likert scales).	Cronbach α coefficient was 0.87 in this study.
**Social closeness**				
	Patient Reported Outcomes Measurement Information System Social Isolation Short Form 4a	[70]	Self-report 4-item scale captures social relationships.	Some of the items focus on domains typically impacted by institutional residence and may influence responses.
	Inclusion of Other in Self Scale	[39,40]	Single-item graphical measure that shows 2 circles for self and others at various levels of distance until they substantially overlap. Participants indicate which one best represents the perceived interaction with the onsite companions.	No comment.
	Quality of the Caregiving Relationship Questionnaire	[67]	Fourteen items with two subscales, warmth and absence of conflict, measuring quality of relationship between participant and relative.	Ceiling effect noted.
	Social Support Behavior Scale	[77]	Three subscales—social support network, quantity of social support, and satisfaction with social support.	No comment.
	Lubben Social Networking Scale	[63]	Self-report measure of the size of social networks with friends and family.	Recommended measure for assessing social networks among older adults.
**Loneliness**				
	UCLA^c^ Loneliness Scale	[77,80,86,96]	Self-report 3-item scale measuring loneliness.	Previous studies have shown this to be a reliable and valid measure of loneliness.
**Agitation**				
	Cohen-Mansfield’s Agitation Scale	[86]	Eight problematic behaviors were coded for frequency of occurrence on a scale of 0=did not occur to 3=occurred almost constantly. These included leaving one’s seat, cursing or verbal aggression, requests for attention, asking repetitive questions or sentences, screaming, shouting or making strange noises, complaining, engaging in physical aggression to self or others, and exhibiting general restlessness.	Interrater reliability was established at >90% agreement among 3 independent coders. Mood prior to the study was observed to have an impact on agitation observed during the study, potentially skewing the results. Baseline agitation levels should be collected as a baseline to allow for comparison.
**Depression and anxiety**				
	Center for Epidemiologic Studies Depression Scale	[75]	Self-report 8-item scale measuring depressive symptoms in the last week.	No comment.
	Cornell Scale of Depression in Dementia	[63]	Clinician rated 19-item scale indexing mood and related signs, behavior disturbance, cyclic function, ideational disturbance, and physical signs of depression.	No comment.
	Geriatric Depression Scale	[63,77,96]	Thirty items with a yes or no response.	The validity of the scale decreases when older adults have cognitive impairment.
	Geriatric Depression Scale—Residential	[67]	A 12-item depression scale relevant to nursing and residential home populations.	No comment.
	Chinese Geriatric Depression Scale	[86]	A 15-item scale measuring feelings of depression over the prior week.	Cronbach α coefficient was 0.90 in this study.
	Geriatric Anxiety Inventory	[63]	A 29-item scale with yes or no responses relating to anxiety over the prior week.	Strong utility in detecting anxiety disorders.
**Psychosocial capacity**				
	Mini-ICF^d^ Rating for Impairment in psychological activities and capacities	[81]	Assesses psychosocial capacity in nursing homes through (1) adherence to regulations, (2) planning and structuring of tasks, (3) flexibility, (4) competence and knowledge application, (5) capacity to make decisions and judgments, (6) proactivity and spontaneous activities, (7) endurance, (8) self-assertiveness, (9) contact with others, (10) group integration, (11) intimate relationships, (12) self-care, and (13) mobility.	The tool is recommended in social medicine guidelines. Proven to be manageable, reliable, and valid in clinical practice.

^a^EuroQOL: EuroQOL consists of a Research Foundation and Group Association, which provides tools for measuring and valuing health that aim to improve decisions about health and health care globally.

^b^DEMQOL: Dementia Quality of Life is a patient-reported outcome measure, which is designed to enable the assessment of health-related quality of life of people with dementia.

^c^UCLA: University of California, Los Angeles.

^d^ICF: International Classification of Functioning, Disability and Health.

Engagement was measured using the Engagement of a Person With Dementia Scale [94], Observational Measurement of Engagement [94], Music in Dementia Assessment Scale [70], Engagement in Meaningful Activities Survey [70], Video Coding Incorporating Observed Emotion protocol [51], the Ethographic and Laban-Inspired Coding System of Engagement [88], and Visual Focus of Attention [103]. Well-being and satisfaction were measured through the Warwick-Edinburgh Mental Well-Being Scale [59], World Health Organization-Five Well-Being Index [81], the Satisfaction With Life Scale [75], and Ryff’s Psychological Well-Being Scale [85]. Emotional response was assessed through the Observed Emotional Rating Scale [38,88], 4 items measuring enjoyment from the Game Experience Questionnaire [39,40], and the People Environment Apathy Scale [94]. Quality of life was measured using the EuroQOL EQ-5D-5 L [80], Multicultural Quality of Life Index [70], Quality of Life in Late Stage Dementia scale [70], the Dementia Quality of Life Proxy instrument [38], and the Quality of Life—Alzheimer’s Disease scale [67]. Purpose and meaning were measured by the Chinese Health Questionnaire [86] and the Purpose in Life Survey [86]. Social closeness was measured using the Patient Reported Outcomes Measurement Information System Social Isolation Short Form 4a [70], the Inclusion of Other in Self Scale [39,40], the Quality of the Caregiving Relationship Questionnaire [67], the Social Support Behavior Scale [77], and the Lubben Social Networking Scale [63]. Loneliness was measured using the University of California, Los Angeles 3-Item Loneliness Scale [80]. Agitation was assessed through the Cohen-Mansfield’s Agitation Scale [51,72]. Depression was measured using the Center for Epidemiologic Studies Depression Scale [75], the Cornell Scale of Depression in Dementia [63], the Geriatric Depression Scale [63,77,96], the Geriatric Depression Scale—Residential [67], and the Chinese Geriatric Depression Scale [86]. Anxiety was measured using the Geriatric Anxiety Inventory [63]. Psychosocial capacity was measured using the Mini-ICF Rating for Limitations of Activities and Participation in Psychological Disorders [81], which includes subscales assessing group integration and intimate relationships.

Instruments included a mixture of self-report scales and observation of behavior. Observational tools were more commonly seen when residents also had a diagnosis of dementia. While some authors did not justify their choice of instruments, others provided justification through the provision of evidence on their validity and reliability in similar contexts. Challenges to the use of the tools were also recorded, including potential inconsistencies between different raters [35,67], the impact of the long-term care setting on the accuracy of the results [70], and the conceptual gaps around interpreting affective facial expressions, connectedness, and social inclusion [51].

### Factors That Support Meaningful Connection

#### Overview

A narrative summary was developed to explore how the included studies reported the factors underpinning meaningful connections during the use of digital technology. Five key themes were identified: getting to know the person, increases autonomy and choice, source of enjoyment and fun, facilitates communication, and builds community. Table 4 identifies the studies associated with each theme.

**Table 4:** Summary of papers associated with factors that support meaningful connections.

Theme and subtheme		Papers
**Getting to know the person**		
	Reminiscence or life story work	[33-36,41,47,49,53,55,57,67,70,71,75,77,78,81-84,87,88,93,94]
	Seeing the patient as a person	[36-38,43,46,55,79,84,86,90]
**Increases autonomy and choice**		
	Autonomy and control	[34,52,56,64,70]
	Personal choice valued	[34,48,58,74,79,81,84,104]
**Source of enjoyment and fun**		
	Technology was fun	[39-41,52,53,55,56,59,64,65,70,73,74,81,88,89,91,92,104]
	Space for creativity	[34,56,70,73,79]
**Facilitates communication**		
	Communicating with a facilitator or HCP^a^	[31,37,46,50,52-54,56-58,63,68,81,88,90,93,96]
	Communicating with family	[32,33,36,38,41,43-47,49,50,52,60-62,67,75-78,93]
**Builds community**		
	Shared social activities within the care home	[39,40,51,52,56,63,64,66,71,74,76,78,81,87,89,93,99-103]
	Intergenerational and wider community connection	[39,40,42,49,50,71,74,75,80,104]

^a^HCP: health care professional.

#### Getting to Know the Person

Reminiscence and life story work were identified as a key mechanism through which meaningful connections were formed by providing an opportunity to get to know the person as an individual without focusing on their medical condition. Reminiscence was enabled through a range of activities from virtual cycling by reminiscing on experiences of cycling earlier in life [83] to VR photo albums and videos [85]. Technology supported less pressured nonverbal interactions, allowing personal preferences to emerge, helping practitioners to get to know individuals more effectively [54], and offering multiple channels through which people could express their identity [52]. Shifting from patient to person was seen not only in reminiscence-specific technologies such as MEMENTO, a spontaneous reminiscence therapy smartwatch [58], but also in the use of robotics such as the social sheep [88,94,95] and in the development of personalized multimedia spaces through life story work such as SENSE-Garden [79]. Residents enjoyed when activities led to an increased feeling of value and a sense of being treated seriously, for example, when combining 3D VR with hands-on horticultural therapy [86]. Getting to know the individual resident led to a change in the care culture, for example, gaining deeper insights into residents’ life stories to facilitate SENSE-Garden fostered a more profound personal connection and shift in focus to a person-centered approach.

#### Increases Autonomy and Choice

Digital technologies provided an opportunity for residents to feel in control, empowered, and independent while having their autonomy and personal choice respected, creating spaces for meaningful connections to form. Many of the technologies, for example, virtual talks [34], SENSE-Garden [79], VR Vacation Home [81], and Virtual Creative Ageing Program [70], all promoted a sense of control and autonomy over the activity, which was reported as a key element that promoted meaningful connections. Computer kiosks provided an opportunity for personal development while being in control, which helped to increase residents’ feelings of self-worth and esteem as well as connecting socially [64]. Lifestyle enrichment was also noted in the study by Webber et al [48], which used technology to facilitate virtual visits to places of personal significance. Residents could also meaningfully connect with their families without needing staff as gatekeepers [50]. Promoting ability instead of focusing on limitations created positive experiences for residents and staff.

#### Source of Enjoyment and Fun

To build meaningful connections, this theme identified the importance of digital technologies as a source of enjoyment and fun for the users. Digital technology was described as playful [53,55,59,89], pleasurable [65], fun [39,40], and enjoyable [52,88,103]. The digital technology created a space to express creativity [70], engage imagination [56], socialize [43], and break routine [34], which relieved stress and boredom for residents. Robotics in particular was identified as a source of shared joy, such as Pepper, the social semihumanoid robot [91], and Paro, the baby harp seal [92].

#### Facilitates Communication

Digital technologies were often implemented with facilitation from a health care professional or study-specific facilitator. The process of engaging with the facilitator was identified as a key opportunity to connect meaningfully, for example, during the delivery of digital cognitive stimulation therapy [31] or the introduction of the interactive sound cushion VITA [53]. Spending more time with staff while engaging with technology was reported positively by residents [68]. Technology supported meaningful communication with the facilitator, enabling trust and reducing misunderstandings [37]. Robotics such as pet cats and dogs provided something positive to talk about between the residents and health care professionals, opening up a channel of communication [96]. In addition to increasing communication with a facilitator, some technologies increased communication with families, for example, teleconferencing [44,45] and video calling relatives [33,41,47,77]. Apps such as Memory Keeper acted as a conversation starter [36], and new devices, such as a digital television for reminiscence [67], VENSTER interactive artwork, encouraged conversation about a present activity [57]. KOMP supported the maintenance of social bonds through family interaction [60,61], and a digital photo frame improved social connectiveness with family [62].

#### Builds Community

Opportunities were created to engage in a social activity, which supported residents to find others with shared interests and develop a sense of community. Meaningful social relationships and small group interactions were enabled through the use of online social networking in the study by Ysseldyk et al [75] and the use of a Computer Interactive Reminiscence and Communication Aid in the study by Samuelsson et al [76]. The introduction of new devices such as an interactive table [51], PARO the baby harp seal [100], virtual visits to museums [39,40], technology-assisted music making [66], and shared activities such as SitDance [74] encouraged interaction between residents, staff, and relatives through a shared social activity, reducing loneliness. VR Take a Cycle With Me supported intergenerational rapport and cohesiveness, which fed into an increased sense of community [80], and adaptive Motitech bikes provided a purpose and challenge while building a sense of community with other residents in an inclusive way [63]. Technology improved participation for people living with dementia, with robotic cats and dogs promoting an inclusive community for all abilities in care [90], zoomorphic robots initiating conversations [99], robot-led activities improving engagement [101], and robot-facilitated dance sessions improving group valence [103].

## Discussion

### What This Study Adds

This scoping review explored the findings from 72 papers that identified the active digital technologies used in care homes to help build meaningful connections, as well as the factors that underpin the formation of meaningful connections and the range of outcome measures used to assess their impact. Based on our search of CINAHL, MEDLINE, and *JBI Evidence Synthesis* up to December 12, 2025, we did not identify any previous scoping review that looks at meaningful connections across the care home, without focusing on a specific condition or clinical outcome. Five main types of digital technology were identified, including mobile apps, VR and immersive technology, robotics, digital devices and ambient technology, and online or computer-assisted programs. A narrative summary described the factors through which meaningful connections were developed, and these included getting to know the person, increases sense of autonomy and choice, source of enjoyment and fun, facilitates communication, and builds community. While most studies relied on some form of qualitative analysis or observation to identify changes in meaningful connections, a wide variety of standardized instruments were also used, which looked for changes in engagement, well-being or satisfaction, emotional response, quality of life, purpose and meaning, social closeness, loneliness, depression and anxiety, and psychosocial capacity.

### Comparison of Findings With Existing Literature

The findings from this scoping review concluded that meaningful connections were facilitated through the creation of opportunities for the residents to be known and heard as a person, and the development of group and shared spaces where people could share an enjoyable experience and an increased sense of community. Effective digital technologies amplified human interactions instead of replacing them, enabling increased interaction with family, friends, and community outside of the care home, shared activities and hobbies with other residents in the care home, and opportunities for staff and facilitators to get to know the resident on a deeper level. These findings align with a community-based implementation project of social robots with older adults, which found that social robots facilitated person-person interactions and inspired participants to share experiences with their family and friends after the engagement session [105]. Fostering and maintaining meaningful connections is at the core of social health, which is defined as an adequate quantity and quality of relationships in a particular context to meet an individual’s need for meaningful human connection [3]. These findings align with wider research into the impact of digital technology on patient experience, which reported improvements in patient experience and improved quality of care [106].

While digital technology plays a key role in addressing social health by acting as a catalyst for forming meaningful human connections, caution is also due when considering the potentially harmful effects of overreliance on artificial intelligence companions that could replace social human interaction [107]. Warm technology, which aims to enhance human potential and social connectedness [108], has been developed primarily within the field of dementia care, but could be further developed and tested for application across the wider care home population. Furthermore, focus should be given to ensuring that both staff and residents are familiar with and comfortable using such technologies [109]. However, it is also important to remember that while many of the technologies included a social element, not all meaningful connections were inherently social. Some technologies such as the MEMENTO smartwatch [58] encouraged companionship through spontaneous meaningful conversations and enhanced staff access to residents’ stories through reminiscence. SENSE-Garden created space for interactions between staff and residents, during which they engaged with life stories, focused on abilities more than limitations, and supported a culture shift from patient to person [79]. This highlights the fundamental importance of technology as a facilitator of individual meaningful connection, as well as a facilitator of social connection and community. While some studies support the integration of technology-driven solutions into caregiving procedures to promote social health among nursing home residents with dementia [102], it is also important to consider the potential practical, clinical, and ethical limitations of the introduction of such technologies. From a practical perspective, research has shown that the use of technology for social connection in aged care requires careful facilitation from staff, better resourcing and infrastructural support, collaborations with volunteers, and more attention to individual needs [110]. Clinically guided, emotional warmth, complex social interactions, and empathy are essential in the care of older adults [111]. This may be reduced or removed if the care sector becomes overreliant on technology to meet social health needs. Ethically, the use of social robots should be guided by the same ethical guidelines followed in all therapeutic interventions [112]; however, it was noted in a recent qualitative analysis of research into robotics in health care of older adults that limited attention is given to ethical and legal aspects of care robots [113].

The application of such active digital technologies to social health sits in contrast to the use of passive technologies, which improve safety and efficiency but may inadvertently reinforce a surveillance-oriented model and add little to the social well-being of residents in care homes [114]. A scoping review of digital technologies supporting aged care services more broadly found 7 key roles of digital technology, including health monitoring and assessment, remote health care services, assistive technology to support treatment, self-care management, social technology to facilitate interaction, clinical decision support, and aged care quality measurement [115]; however, only social robots to provide better communication were discussed. When looking at policies focused on digital health, the core messages around technology use in health care move toward clinical applications, such as diagnosis, decision-making, virtual care, remote monitoring, remote data capture, and clinical trials within the Global Strategy on Digital Health 2020-2025 [116]. The focus is once again on more passive clinical monitoring than the application of active digital technology to social well-being. This trend continues in new research on generative artificial intelligence (GenAI), which, despite the capacity to address social health concerns, is currently focused on increasing task efficiency such as scheduling and care planning [117]. Alternatively, active technologies have a greater potential to promote engagement and connection [118,119]. While their effectiveness is often dependent on family or professional caregivers to help set up and use the technology to ensure meaningful use rather than superficial engagement [120], the involvement of caregivers is, in itself, a mechanism through which more meaningful connections can be achieved.

The scoping review showed how the types of digital technologies used for meaningful connection have changed over time and have exponentially increased in the last 3 years. Smart Home technologies have been shown to significantly enhance safety and monitoring capabilities for adults living independently [121]; however, this overlooks the social health of older adults. A current area for future exploration is the application of GenAI in care homes to promote meaningful connections. GenAI, while originally appearing in physical form through the chatbot Eliza in the 1960s, has recently boomed in popularity in the public sphere through chatbots such as ChatGPT, and more recently again through its application to health care [122]. Currently, the primary application in clinical practice is to support health care, planning, and information searching [117]. For example, one study successfully applied GenAI to reduce emergency incidents and help daily activities run more smoothly within assisted living environments [123]. Another study explored how GenAI models can improve decision accuracy among emergency nurses; however, they noted the lack of contextual sensitivity in emergency care [124]. Within nursing practice, GenAI is being embedded in patient care, supporting workflow integration, and providing operational support [125]. In gerontological nursing education, GenAI has been integrated with art therapy to support the development of empathy in students [125]. In community care, artificial intelligence–powered reflection tools have been shown to improve communication between older adults and caregivers [126], to support older adults with health information tasks [127,128], and to provide empathetic responses to online queries related to Alzheimer disease and related dementias [129].

However, GenAI with the goal of building meaningful connections has received little attention, specifically in the long-term care setting, which has additional barriers to meaningful connections. The HARMONEE (Harnessing Artificial Intelligence Resources for Mental Well-Being for Older Adults and Nurturing Empathy in Education) project is a novel example of how GenAI can be used to support reminiscence with older adults in long-term care to improve their mood [119]; however, its dependence on a research team to facilitate generates issues with the sustainability. Technology must be designed so it can be used by the resident or care team independently. Additionally, to ensure that technology is fit for purpose and suited to the unique setting of the care home, care home residents must be part of decision-making when it comes to co-designing and implementing new technologies in order to defy techno-solutionist scripting of dehumanizing images of fragility, dependency, and technical ineptitude embedded in product designs [130]. Research has indicated an openness of older adults to new technologies for physical assistive functions [131]; however, further exploration is needed to understand their attitudes toward technologies aimed at supporting meaningful connections.

This review identified that the measurement of meaningful connection was inconsistent across studies. This may be attributable to the varying operational definitions of meaningful connections, activities, and engagement across these studies. Qualitative analysis and observation were most frequently used to assess concepts associated with meaningful connections. Studies that used scales and instruments considered a wide variety of measures including engagement, well-being or satisfaction, emotional response, quality of life, purpose and meaning, social closeness, loneliness, depression and anxiety, and psychosocial capacity. This highlights a broader challenge around defining and measuring meaningful connections in the literature. It could be argued that meaningful connections in themselves are not measured specifically, but instead outcome measures focused on indicators of meaningful connections (eg, well-being, loneliness, and quality of life). When exploring social connection, Holt-Lunstad [2] discusses similar terminological challenges; for example, loneliness is often used to measure social connection; however, it is defined as a distressing experience resulting from perceived inadequate meaningful connections; therefore, it does not fully capture the breadth of social connection. Similarly with meaningful connections, a clear consensus has not been reached on a definition or approach to measurement. Measurement of social connection is being progressed, for example, through the development of the Connection During Conversations Scale [132]; however, with the focus being on social connection during social interactions, it is possible that it overlooks some of the meaningful connections occurring outside social spaces.

### Strengths and Limitations

By adopting a scoping review approach, a comprehensive overview of the broad field of digital technology within care homes could be provided, helping to explore the scope of existing knowledge and identify gaps in the evidence base to highlight for future research. The scoping review was conducted with strict adherence to the JBI methodology and included multiple authors at each stage to minimize the risk of individual bias. However, limitations are also noted. While the language of meaningful connections was left as broad as possible within the search strategy, it is possible that papers with relevant information around social connection or person-centered care delivery were missed. Given the time and resource restraints within the project, the decision was made to limit the search to the English language. However, language bias may result in missing important cultural contexts in non-English–speaking settings, limiting the relevance of findings in some geographical locations. The main focus of this review was on active digital technologies, excluding passive monitoring technologies. However, these technologies contribute to care home life through their influence on care practices, staff time and availability, and safety perceptions that directly impact opportunities for connection. It is possible that passive monitoring technologies could increase staff availability and create a safer care environment, which would lead to increased opportunities for meaningful connections. Through their omission from the review, only a partial understanding of the digital landscape within care homes has been explored. Finally, the heterogeneity of outcome measures reported across the studies limits the comparability of studies and restricts the synthesis of these findings. Clear conclusions regarding the measurement of meaningful connections could not be drawn; however, this did highlight a gap for future research.

### Recommendations and Real-World Implications

At present, technologies used within aged care seem to not be meeting end user needs [133]. While technology design for older adults is often focused for family members or caregivers to allow passive monitoring, giving control back to older adults nurtures their sense of autonomy [134]. Future technologies must also be designed with social health in mind, considering both the engagement of the individual and the community. This review has highlighted how technology can facilitate both individual meaningful connections as well as social connections and a sense of community. However, to ensure technology is fit for purpose and suited to the unique setting of the care home, care home residents must be part of decision-making when it comes to co-designing and implementing new technologies in order to defy the dehumanizing images of fragility, dependency, and technical ineptitude embedded in product designs [130]. Furthermore, future research needs to consider the cultural environment of the care home when embedding new active digital technologies with the goal of building meaningful connections, as research has shown that how the cultural environment has the most significant influence on nursing home residents’ leisure and social occupational choice [135].

Future research needs to consider the development and psychometric testing of a standardized tool to measure meaningful connection more broadly, as well as in relation to the digital technologies implemented. Consideration needs to be given to both individual and social connection indicators, as well as emotional response, engagement, satisfaction, loneliness, anxiety, depression, and quality of life. There is also a gap specifically around meaningful connections within care homes. The challenge raised with some of the instruments used to collect outcome measures was the uniqueness of the care home setting. Challenges in fostering meaningful engagement in care homes include a lack of understanding of residents’ values and interests, staff needing to manage multiple requirements simultaneously, and staff not receiving support to involve residents [136]. Some of the items in the scales were not reflective of, or relevant to, long-term care settings, leading to the need for modifications or the acknowledgment that this may have impacted on the findings of the study. Future research needs to consider the development of a bespoke instrument that considers meaningful connections within the context of the care home environment.

Research in this space is rapidly expanding, aligned with advances in digital technology availability. However, few studies that focused on meaningful connections considered an implementation design. The reliance on qualitative and observational methods may link to the challenges in measurement or the infancy of the technology; however, future research needs to consider the development of implementation studies, considering broader factors such as cost-effectiveness and organizational readiness. The TIDieR (Template for Intervention Description and Replication) checklist [137] may structure the reporting of pre- and postinterventions from other settings, allowing them to be replicated in the care home context.

### Conclusions

This scoping review has presented a detailed exploration of how digital technologies have been used within care homes to facilitate the development of meaningful connections between residents, families, staff, and communities. While the wider literature indicates that many digital technologies overlook social health and well-being in care homes, this review highlights how a range of technologies including mobile apps, VR and immersive technology, robotics, digital devices and ambient technology, and online or computer-assisted programs can be applied to facilitate meaningful connections within care homes. Digital technology is a catalyst for meaningful human connection, which is a key element of social health, rather than a replacement. Further research is needed to explore how digital technologies can support individual meaningful connections with residents as well as enhance the sense of community through shared social activities and spaces. The application of GenAI to the development of meaningful connections and social health in care homes is a current gap in the evidence base. Moving forward, the development of GenAI-based technologies needs to be undertaken in conjunction with care home residents, staff, and communities to ensure that they meet the social needs of the entire community. An ongoing challenge is found in the measurement of the effectiveness of digital technologies to enhance meaningful connections. Future research needs to consider how new evaluation metrics can be developed that combine digital engagement data with validated measures of social and meaningful connection to assess the person-centered impact of digital technologies in care homes.

## Appendix

Multimedia Appendix 1 PRISMA ScR checklist.

Multimedia Appendix 2 Search strings per database from search on December 12, 2025.

Multimedia Appendix 3 Data summary table.

## Abbreviations

GenAI: generative artificial intelligence
HARMONEE: Harnessing Artificial Intelligence Resources for Mental Well-Being for Older Adults and Nurturing Empathy in Education
JBI: Joanna Briggs Institute
MeSH: Medical Subject Headings
PRISMA-S: Preferred Reporting Items for Systematic Reviews and Meta-Analyses Literature Search Extension
PRISMA-ScR: Preferred Reporting Items for Systematic Reviews and Meta-Analyses Extension for Scoping Reviews
TIDieR: Template for Intervention Description and Replication
VR: virtual reality
WHO: World Health Organization

## Data Availability

All data generated and analyzed during this study are included in this published paper and Multimedia Appendix 3.
