# Supplementary material for: Digital Technologies That Support Meaningful Connections in Care Homes: Scoping Review
Source: J Med Internet Res. 2026 Mar 3;28:e88181. doi: 10.2196/88181 (PMC12996902; doi:10.2196/88181)
Supplement: Multimedia Appendix 2 [file jmir_v28i1e88181_app2.docx]

| Database | Concept 1 (#) | Concept 2 (#) | Concept 3 (#) | Additional Limiters (#) | Hits |
| --- | --- | --- | --- | --- | --- |
| CINAHL | meaningful or meaning* (96,588) | MH "Nursing Homes+" or "residential care" or "care home*" or "long term care" or “long-term care” or "nursing home*" or “aged care” or "residential home*" (13,703) | MH "Digital Technology+" OR MH "Digital Health+" or "digital technolog*" or digital* or technolog* or "virtual reality" or video* or record* or tablet* or "social media" or "smart phone*" or "mobile phone*" or internet or "social network*" (926,611) | Language (English) | 415 |
| Medline OVID | meaningful or meaning* (214120) | Nursing Homes/ or "Homes for the Aged"/ or Residential Facilities/ or "residential care" or "care home*" or "long term care" or “long-term care” or "nursing home*" or “aged care” or "residential home*" (112,432) | Nursing Homes/ or "Homes for the Aged"/ or Residential Facilities/ or "residential care" or "care home*" or "long term care" or “long-term care” or "nursing home*" or “aged care” or "residential home*" (3,121,538) | Language (English) | 318 |
| PsycInfo | meaningful or meaning* (219,440) | Nursing Homes/ or Residential Care Institutions/ or Elder Care/ or "residential care" or "care home*" or "long term care" or “long-term care” or "nursing home*" or “aged care” or "residential home*" (48,187) | Digital Technology/ or Electronic Health Services/ or Internet/ or "digital technolog*" or digital* or technolog* or "virtual reality" or video* or record* or tablet* or "social media" or "smart phone*" or "mobile phone*" or internet or "social network*" (5,627,027) | Language (English) | 1960 |
| Scopus | meaningful or meaning* (2,433,454) | “residential care" or "care home*" or "long term care" or “long-term care” or "nursing home*" or “aged care” or "residential home*" (580,801) | "digital technolog*" or digital* or technolog* or "virtual reality" or video* or record* or tablet* or "social media" or "smart phone*" or "mobile phone*" or internet or "social network*" (40,636,952) | Language (English)  Subject (Human) | 9196 |
| IEEE XPlore | meaningful or meaning* (30,484) | “Nursing Home*’’ or “Care Home*” or “Residential Home*” or “Residential Care” or “Long-term care*” (556) | “digital technolog* or digital*” or technolog* or “virtual reality” or video* or record* or tablet* or “social media” or “smart phone*” or “mobile phone*” or internet or “social network*” (869) |  | 47 |
| ACM Digital | meaningful or meaning*  (122,011) | "nursing home*" OR "Care home*" OR "residential home*" OR "residential care*" OR "long-term care" OR "long term care" (269,786) | AND "digital technlog*" OR "digital*" OR "technolog*" OR "virtual reality" OR "video*" OR "record*" OR "tablet*" OR "social media" OR "smart phone*" OR "mobile phone*" OR "internet" OR "social network*"(686,571) |  | 785 |
